# Proteomic-based identification of haptoglobin-1 precursor as a novel circulating biomarker of ovarian cancer

**DOI:** 10.1038/sj.bjc.6601882

**Published:** 2004-06-15

**Authors:** N Ahmed, G Barker, K T Oliva, P Hoffmann, C Riley, S Reeve, A I Smith, B E Kemp, M A Quinn, G E Rice

**Affiliations:** 1 Gynaecological Cancer Research Centre, Royal Women's Hospital, 132 Grattan Street, Carlton, Victoria 3053, Australia; 2 St Vincent Institute of Medical Research, 9 Princess Street, Fitzroy, Victoria 3065, Australia; 3 Baker Heart Research Institute, St Kilda Road, Australia

**Keywords:** proteomics, biomarkers, ovarian cancer, haptoglobin, haptoglobin-1 precursor (HAP1)

## Abstract

Screening for specific biomarkers of early-stage detection of ovarian cancer is a major health priority due to the asymptomatic nature and poor survival characteristic of the disease. We utilised two-dimensional gel electrophoresis (2DE) to identify differentially expressed proteins in the serum of ovarian cancer patients that may be useful as biomarkers of this disease. In this study, 38 ovarian cancer patients at different pathological grades (grade 1 (*n*=6), grade 2 (*n*=8) and grade 3 (*n*=24)) were compared to a control group of eight healthy women. Serum samples were treated with a mixture of Affigel-Blue and protein A (5 : 1) for 1 h to remove high abundance protein (e.g. immunoglobulin and albumin) and were displayed using 11 cm, pH 4–7 isoelectric focusing strips for the first dimension and 10% acrylamide gel electrophoresis for the second dimension. Protein spots were visualised by SYPRO-Ruby staining, imaged by FX-imager and compared and analysed by PDQuest software. A total of 24 serum proteins were differentially expressed in grade 1 (*P*<0.05), 31 in grade 2 (*P*<0.05) and 25 in grade 3 (*P*<0.05) ovarian cancer patients. Six of the protein spots that were significantly upregulated in all groups of ovarian cancer patients were identified by nano-electrospray quadrupole quadrupole time-of-flight mass spectrometry (n-ESIQ(q)TOFMS) and matrix-assisted laser desorption ionisation time-of-flight mass spectrometry (MALDI-TOFMS) as isoforms of haptoglobin-1 precursor (HAP1), a liver glycoprotein present in human serum. Further identification of the spots at different pathological grades was confirmed by Western blotting using monoclonal antibody against a haptoglobin epitope contained within HAP1. Immunohistochemical localisation of HAP1-like activity was present in malignant ovarian epithelium and stroma but strong immunostaining was present in blood vessels, areas with myxomatous stroma and vascular spaces. No tissue localisation of HAP1-like immunoreactivity was observed in normal ovarian surface epithelium. These data highlight the need to assess circulating concentration of HAP1 in the serum of ovarian cancer patients and evaluate its potential as a biomarker in the early diagnosis of ovarian cancer.

Epithelial ovarian cancer is associated with a high mortality rate despite the high cure rate for early-stage disease by surgical resection alone. This discrepancy between the high mortality rate and cure at an early stage occurs predominantly due to advanced stage presentation due to lack of symptoms at an early stage (Greenlee *et al*, 2001). Approximately 75% of women present with ovarian cancer at stage 3, when the cancer has spread beyond the pelvis and 5-year survival is only 29% (Landis *et al*, 1999). Over the past 10 years considerable progress has been made in optimising the adjuvant therapy, but these advancements have resulted in modest improvement in disease-free and overall survival. Yet, the mortality from ovarian cancer can be significantly reduced by the application of an effective screening technique at an early stage. To date, despite extensive research and evaluation, the combination of physical examination, imaging with transvaginal ultrasound, and detection and monitoring of the disease using serum marker CA 125 have not resulted in acceptable sensitivity of early-stage ovarian cancer screening. Hence, to save lives from ovarian cancer, there is an urgent need to identify new markers in the serum of early-stage patients.

The serum CA 125 antigen concentration is the ‘gold standard’ for ovarian cancer tumour markers (Lloyd and Yin, 2001). Although this protein was identified 20 years ago its structure and function has not been elucidated (Jacobs *et al*, 1999). With a cutoff of 30–35 U ml^−1^, the sensitivity for CA 125 for early-stage ovarian cancer is 50–60%. Moreover, CA 125 concentrations are elevated in women with benign gynaecologic conditions, including ovarian cysts, endometriosis and uterine fibroids, which are part of the differential diagnosis for ovarian cancer (Mogensen, 1992; Cannistra, 1993; Mackey and Creasman, 1995). In some cases, women with hepatic disease, renal failure, pancreatitis or other conditions may have elevated CA 125 making the usefulness of CA 125 as a marker for early detection limited (Bastani and Chu, 1995; Devarbhavi *et al*, 2002). In recent years, several serum markers have been identified and explored as possible adjuncts to CA 125 screening. Unfortunately, these markers, including CA19-9 and lysophosphatidic acid, have not shown to be clinically relevant in large screening trials (Woolas *et al*, 1999).

Proteomics is a new and emerging technology that can identify protein molecules in a high-throughput discovery approach in patient's serum, biofluids and tissues providing information about which proteins are secreted or released from tumour cells at sufficient concentrations. Recently, the serological proteomic pattern of the ovarian cancer patients has been described that discriminates cancerous from noncancerous groups with a positive predictive value of 94% (Petricoin *et al*, 2002). This approach represents a novel direction in the search for biomarkes for early-stage screening of ovarian cancer wherein a distinct profile of proteins from early-stage cancer patients can create a discriminatory pattern of proteins that can be used as a diagnostic standard. Alternatively, a small subset of differentially expressed proteins from a large volume of profiling data can be identified and these proteins can serve as biomarkers and can be used as targets for further development in molecular diagnostics and therapeutics. Electrospray ionisation mass spectrometry, matrix-assisted laser desorption ionisation time-of-flight mass spectrometry (MALDI-TOFMS) and surface-enhanced laser desorption ionisation time-of-flight mass spectrometry (SELDI-TOFMS) technology have the potential to identify patterns or changes in thousands of proteins and can globally analyse almost all small molecular weight proteins in complex solutions such as serum or plasma.

The aim of the present study was to identify proteins differentially expressed in the serum of ovarian cancer patients at different pathological grades. Proteins found differentially expressed in the serum of ovarian cancer patients after the depletion of high abundance proteins (including albumin and immunoglobulins) were analysed and compared to normal serum using proteomic approaches. A subset of common proteins significantly elevated in grade 1, 2 and 3 of cancer patients were identified by nano-electrospray quadrupole quadrupole time-of-flight mass spectrometry (n-ESIQ(q)TOFMS) and MALDI-TOFMS as a series of haptoglobin-1 precursor (HAP1). This method not only provides efficient profiling of low abundance differentially expressed serum proteins in cancer patients but also allows proteomic-based identification of circulating proteins that have the potential to be used as early-stage markers.

## MATERIALS AND METHODS

This study was approved by the Royal Women's Hospital, Melbourne, Research and Human Ethics Committee (Human Ethics Committee # 02/29 and 02/30). Human blood was collected from healthy volunteers (*n*=8) and patients (*n*=38) presenting at the Oncology/Dysplasia Unit, Royal Women's Hospital, Melbourne, after the provision of a participant information statement and with informed consent. The mean age of women in the control group and women with ovarian cancer was 47 and 62 years, respectively. Of the six patients with grade 1 ovarian cancer four had stage 1, one stage 2 and one had stage 3 disease (Table 1

**Table 1:** Description of ovarian cancer patients participating in the proteomic study

**Case no.**	**Histological grade**	**Clinical stage**	**Tumour subtype**	**CA 125 (U ml^−1^)**
23	1	2B	Endometrioid	27
24	1	3C	Mucinous	281
57	1	1A	Endometrioid	37
98	1	1B	Endometrioid	786
121	1	1C	Endometrioid	NA
2	1	1C	Mucinous	93
11	2	2B	Serous	172
28	2	3C	Endometrioid	93
42	2	3C	Endometrioid	12
54	2	1C	Endometrioid	711
73	2	1C	Endometrioid	15
76	2	3C	Serous	681
86	2	3C	Serous	333
183	2	3C	Serous	3471
3	3	3C	Endometrioid	307
31	3	3C	Endometrioid	996
40	3	4	Endometrioid	3571
51	3	3C	Endometrioid	10384
77	3	3C	Endometrioid	56
108	3	2C	Endometrioid	93
116	3	3C	Endometrioid	255
148	3	3C	Endometrioid	883
160	3	3C	Endometrioid	61
168	3	3C	Endometrioid	137
19	3	3B	Serous	126
20	3	3C	Brenner tumor	123
39	3	3C	Serous	682
83	3	2B	Endometrioid	165
103	3	3C	Clear cell carcinoma	833
122	3	3C	Endometrioid	2112
156	3	3C	Endometrioid	659
163	3	3C	Serous	177
164	3	4	Serous	NA
172	3	3C	Serous	1308
182	3	2C	Serous	NA
184	3	3C	Serous	353
185	3	3C	Serous	682
196	3	3C	Serous	5437

NA=not available.

). Two patients with stage 1, one with stage 2 and five with stage 3 ovarian cancer were included in grade 2 group. All patients, except two with grade 3 ovarian cancer had stage 3 disease. The remaining two patients in grade 3 were diagnosed with stage 4 ovarian cancer (Table 1).

Ovarian cancer patients with serous, mucinous, endometrioid and clear-cell carcinoma subtypes were included in the study (Table 1). One patient diagnosed with Brenner tumour was also included in the study (Table 1). Of the six patients in grade 1 group, four were diagnosed with endometrioid tumour and the other two had mucinous tumours. Grade 2 patients comprised of four serous and four endometrioid subtypes. Nine serous and 13 endometrioid subtypes of patients were included in grade 3 subgroup. One clear-cell carcinoma and one Brenner tumour subtype was also included in the group. Serum samples were collected from patients after diagnoses and before surgery. All ovarian cancer patients except three had CA 125 values above the cutoff limit of 35 U ml^−1^ (Table 1). All patients except two in grade 1 group with a clinical diagnoses of stage 1 and 2 and one grade 3 patient at stage 3 presented with ascites.

Whole blood (10 ml) was collected by venepuncture into plain collection tubes for serum (blood was allowed to clot at room temperature for 30 min). Samples were centrifuged at 2000 **g** for 10 min after which serum was collected. An aliquot (100 *μ*l) was removed for the determination of total protein. Serum was stored at −80°C until analysed.

### Protein assay

Total protein content was determined using a commercial protein assay kit with BSA standards according to the manufacturer's instruction (Pierce, Rockford, IL, USA).

### Affi-gel Blue and protein A treatment

Human serum samples were treated with a mixture of Affigel-Blue and protein A (5 : 1) in the form of a spin column. The spin columns containing a mixture of Affi-Gel Blue and Protein A selectively bind and remove albumin and immunoglobulin (Ahmed *et al*, 2003). The spin columns were washed twice with 1 ml of binding buffer (20 mM phosphate buffer, pH 7.0) by centrifugation for 20 s at 1000 **g**. In all, 50 *μ*l of serum was added to 150 *μ*l of binding buffer and mixed by vortex and loaded on the spin columns. Following incubation at room temperature for 1 h, columns were centrifuged for 20 s at 1000 **g** to collect the eluate. The columns were washed with 200 *μ*l of the binding buffer and combined with the first eluate to form the depleted serum sample. The total protein concentration of the combined eluate was determined. The eluate was stored at −80°C until further analysis.

### Two-dimensional electrophoresis

#### First-dimension separation

In total, 25 *μ*g of serum protein was mixed with rehydration buffer (7 M urea, 2 M thiourea, 100 mM DTT, 4% CHAPS, 0.5%. carrier ampholytes pH 4–7, 0.01% BPB and 40 mM Tris) to a final volume of 200 *μ*l and incubated for 1 h at room temperature. This mixture was then applied to a Ready Strip® (11 cm, pH 4–7, Bio-Rad Laboratories, USA) and actively rehydrated at 50 V at 20°C for 16 h. Serum proteins were isoelectrically focused at 250 V for 15 min and then slowly ramped up to 8000 V for 150 min and then maintained at 8000 V for a total of 35 000 Vh gel^−1^. (i.e. a total of 42 000 Vh per gel). Ready Strips were then stored at −80°C until second-dimension processing.

#### Second-dimension separation

Ready Strips from the first-dimension separation were equilibrated in 5 ml of equilibration buffer (50 mM Tris-HCl pH 8.8, 6 M urea, 30% glycerol, 2% SDS, 0.01%. BPB, 2 mM tributyl phosphine (TBP)). Strips were rinsed in Tris glycine SDS running buffer (25 mM Tris, 192 mM glycine, 0.1% w v^−1^ SDS pH 8.3) and then applied to the top of a 10% Tris-HCl Precast Criterion Gel (Bio-Rad Laboratories, USA). Low melting point agarose (0.5% in running buffer containing BPB) was layered on top of the strip. Molecular weight markers were run simultaneously. Electrophoresis on the gel was carried out at 10 mA gel^−1^ for 1 h, 20 mA gel^−1^ for 2 h and then 30 mA gel^−1^ for 30 min. Gels were then fixed in methanol/acetic acid (40%/10% in dH_2_O) for 1 h at room temperature and then incubated in SYPRO Ruby® (Bio-Rad laboratories, USA) for 16 h at room temperature on a rocking platform. Gels were destained for 1 h in methanol/acetic acid (10%/7% in dH_2_O), imaged using a Bio-Rad FX imager at 100 nm resolution and analysed using PDQuest version 6. The computer program identified protein spots from the digital images of the gel. Some serum samples were repeated three times to assess the variability between the experiments on different gels.

#### Mass spectrometry

Coomassie-stained proteins were excised from gel, and then digested with trypsin. Mass spectrometry analyses were preformed on an Ettan MALDI-TOF (Amersham Bioscience, UK) and API QSTAR Pulsar i Mass spectrometer (Applied Biosystems, MDS Sciex, Framingham, USA). TOFMS data were searched via PepSea Server, which is included in the Analyst Software (Applied Biosystems, MDS Sciex, Framingham, USA). Tandem MS data were searched via the Mascot search engine.

### Western blotting

Serum samples separated by first and second dimension as described earlier were transferred to nitrocellulose membranes. Membranes were probed with anti-haptoglobin (mouse monoclonal, Sigma, St Louis, USA) followed by peroxidase-labelled secondary antibody and visualised by the ECL (Amersham, UK) detection system according to the manufacturer's instructions.

### Immunohistochemistry

Paraffin processed archival tissues were obtained from the Department of Pathology, Royal Women's Hospital, Melbourne. These included normal ovaries (*n*=6) needed for control comparisons, which were removed from patients undergoing surgery as a result of suspicious ultrasound images, palpable abdominal masses and family history. The pathology diagnosis and tumour grade was determined by two staff pathologists in the Department of Pathology, Royal Women's Hospital, Melbourne. The classification of the tumours was carried out as part of the clinical diagnosis. Histological grading of ovarian carcinoma was carried out by the method described by Silverberg (2000).

Tissue sections were cut at 4 *μ*m thickness, mounted on Poly-L-lysine-coated slides and incubated for 1 h at 60°C. Sections were brought to water through three changes each of xylene and ethanol. Antigen unmasking was undertaken using citrate buffer (pH 6.0) in a microwave oven. Endogenous peroxidases were removed using 3% hydrogen peroxide in methanol and endogenous biotin activity was blocked using a sequence of diluted egg white (5% in distilled water) and diluted skim milk powder (5% in distilled water). Sections were incubated for 1 h in haptoglobin monoclonal antibody (Sigma, St Louis, USA) diluted 1 : 10 000 in 1% BSA in Tris buffer (100 mM pH 7.6). Antibody binding was amplified using biotin and streptavidin HRP (DAKO, Denmark) for 15 min each and the complex was visualised using diaminobenzidine (DAB). Nuclei were lightly stained with Mayer's haematoxylin. An isotype IgG1, suitably diluted, was substituted for the antibody as a negative control.

Sections were assessed microscopically for positive DAB staining. The extent of haptaglobin expression was scored as 0 (<10%), 1 (11–25%), 2 (26–50%), 3 (51–75%), 4 (76–90%) and 5 (>90%). In addition to the extent of staining, tissue and cellular distribution of staining was determined.

## RESULTS

### Removal of high abundant proteins from human serum

Figure 1A

**Figure 1:**
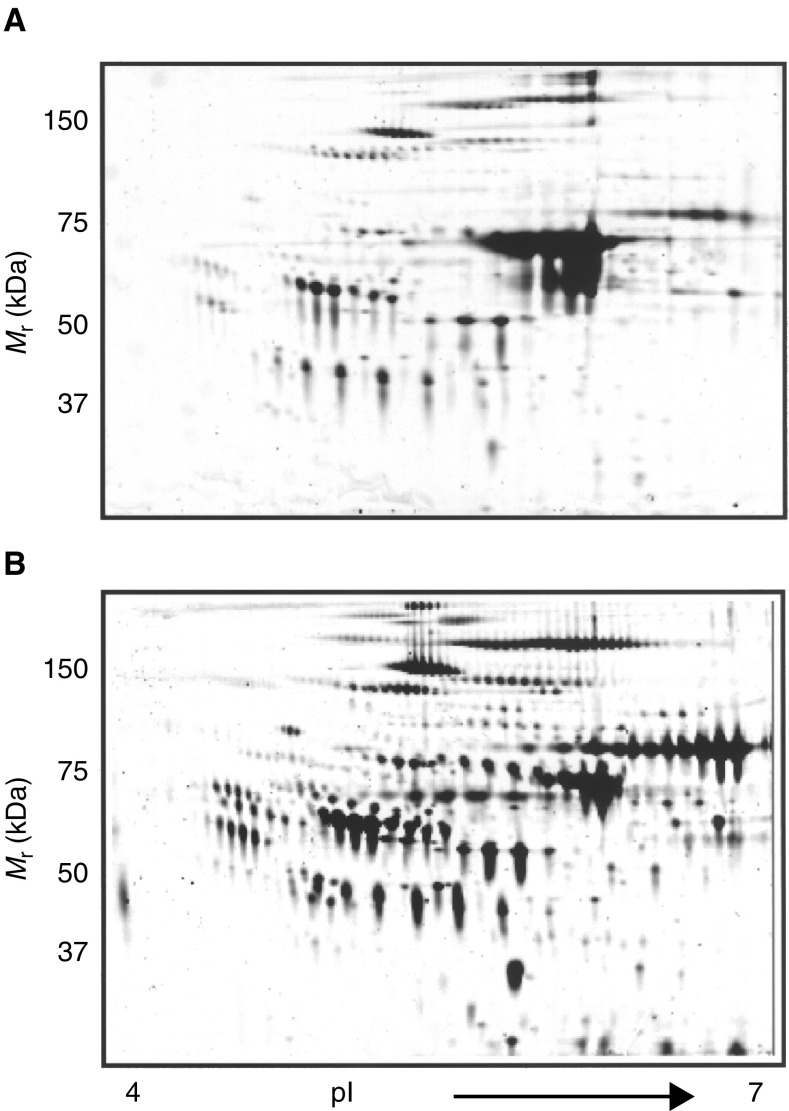
Two-dimensional gel electrophoresis profile of (**A**) untreated serum and (**B**) Affi-Gel Blue and protein A-treated human serum. Human serum was treated with Affi-Gel Blue and protein A (5 : 1) on a column for 1 h before analysis by 2-DE. Protein (50 *μ*g) was loaded on each gel. Results are representative of three independent experiments.

demonstrates a typical two-dimensional gel electrophoresis (2DE) human serum profile evidenced by SYPRO-Ruby staining. More than 300 proteins were detected and localised between pI 4–7 and molecular mass range of 20–200 kDa. The albumin smear at around 68 kDa was present in untreated serum but within 1 h of Affi-Gel Blue and protein A treatment, significant removal of albumin was achieved with no apparent significant loss of other proteins displayed (Figure 1). Concomitant with the removal of albumin there was a significant enhancement in the staining intensity of several protein spots (Figure 1B). These results suggest that Affi-Gel Blue and protein A treatment of human serum results in the removal of high abundance albumin, thereby increasing the detection of low abundance proteins that in the presence of albumin would have remained obscured. We have implemented this approach of albumin clearance for the identification of differentially expressed low abundance proteins in the serum of ovarian cancer patients.

### Serum protein profile of ovarian cancer patients at different histological grades

**Figure 2:**
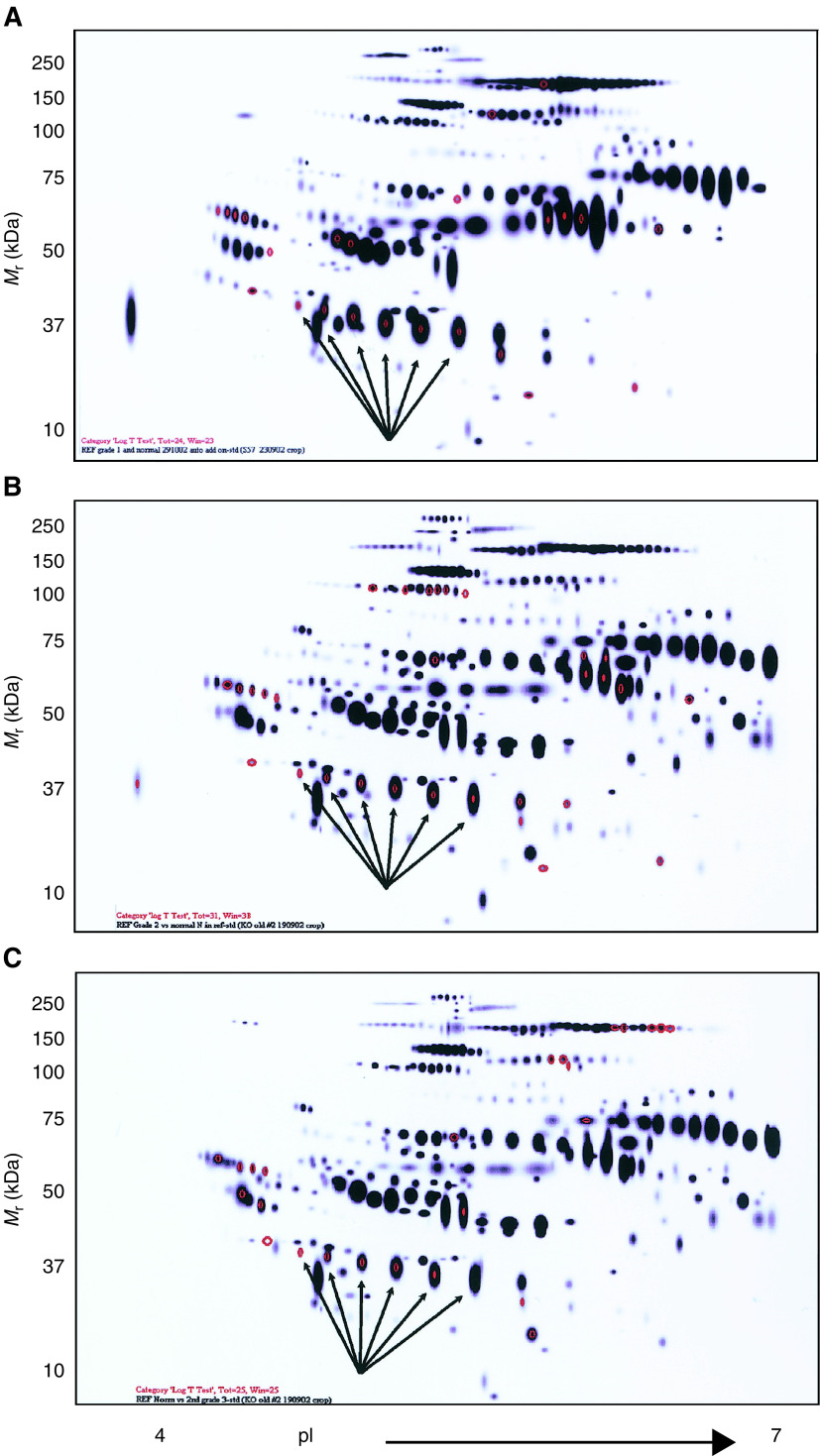
Depiction of the reference profile of all protein spots identified by 2-DE. Profile of proteins differentially expressed (in red) in the serum of (**A**) grade 1 (*n*=6), (**B**) grade 2 (*n*=8) and (**C**) grade 3 (*n*=24) ovarian cancer patients. Comparison between normal (*n*=8) and each pathological grade of cancer patients’ serum was made on replicate sets using PDQuest software.

Protein profiles on the serum of grade 1 (*n*=6), grade 2 (*n*=8) and grade 3 (*n*=24) ovarian cancer patients were analysed by 2-DE and visualised by staining with SYPRO-Ruby. These profiles on replicate sets were compared with the serum of normal healthy women (*n*=8) using PDQuest software with the representation of the Gaussian profile shown in Figure 2A–C

. The quantitative evaluation of the differentially expressed serum proteins in normal *vs* grade 1, grade 2 or grade 3 ovarian cancer patients was performed by using Student's *t*-test. Significant differences in the overall profiles of serum proteins were obtained in grade 1, 2 and 3 ovarian cancer patients compared to normal healthy volunteers. Compared to normal serum, twenty-four proteins were differentially expressed in the serum of grade 1 ovarian cancer patients (Figure 2A). Of these proteins, 15 proteins were upregulated by two-fold, four proteins by five-fold and two proteins by 10-fold. In contrast, one protein was downregulated by two-, five- and 10-fold, respectively. In grade 2 cancer patients, differential expression of 31 proteins was observed of which 25 were upregulated by two-fold, four by five-fold and two by 10-fold. Analysis of serum from grade 3 cancer patients demonstrated two-fold downregulation of 13 proteins out of 25 differentially expressed proteins (Figures 2B and C). There was upregulation of six proteins by two-fold, three by five-fold and two by 10-fold, respectively. Among the differentially expressed serum proteins in the three pathological grades, 10 common proteins were consistently differentially expressed in grade 1, 2 and 3 cancer patients (*P*<0.05). Not unexpectedly, some proteins were found to be uniquely expressed only in the serum of specific pathological grade of cancer patients and were not consistently expressed among the three histological grades of cancer patients. To ensure consistency in the observed differential expression profile, serum samples from the same patient prepared on three different days was repeated three times and was investigated to eliminate confounding factors that may arise from sample handling. No substantial variation in the profile of protein spots of the same sample repeated on different days was detected.

### Protein identification

**Figure 3:**
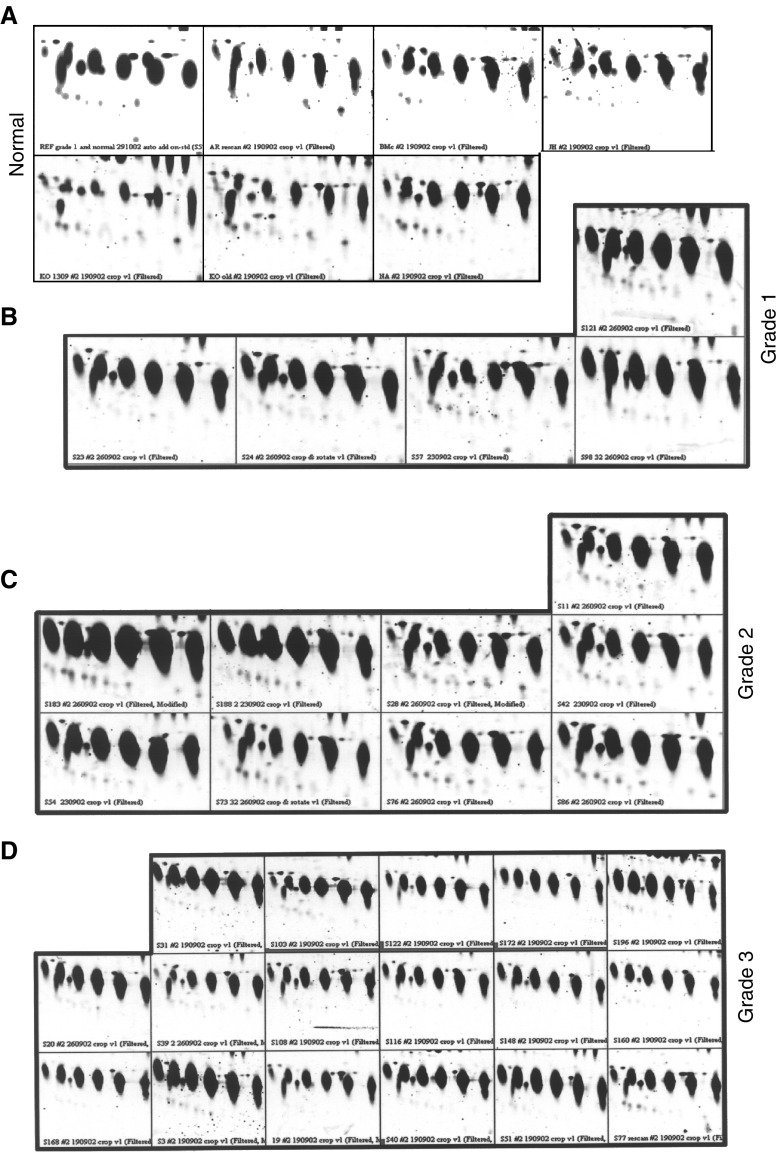
Elevated expression of protein spots 1–6 in the serum of ovarian cancer patients compared to serum from healthy volunteers. Image analysis was performed by using PDQuest software. (**A**) normal serum, (**B**) grade 1, (**C**) grade 2 and (**D**) grade 3 ovarian cancer patients.

**Figure 4:**
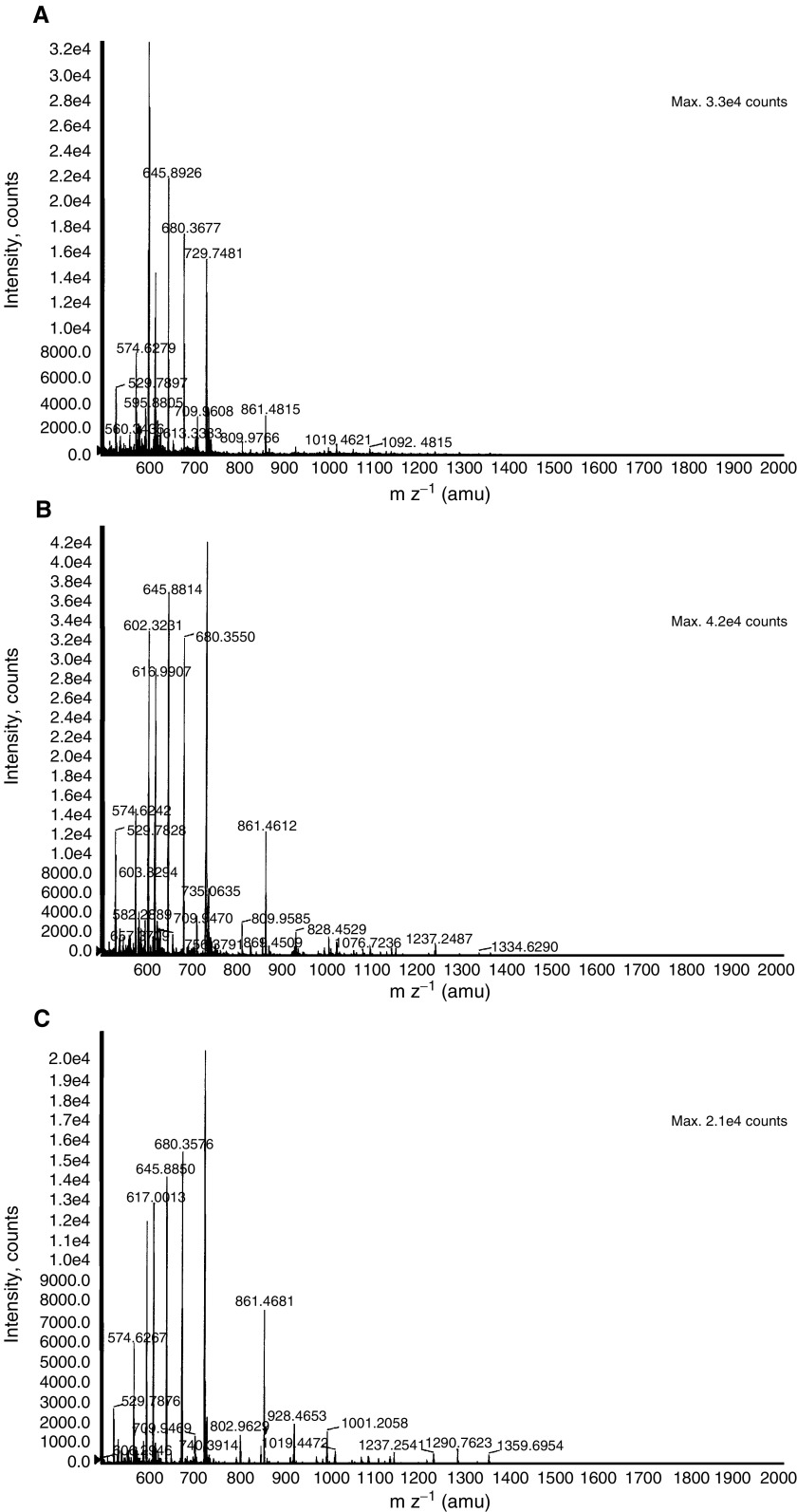

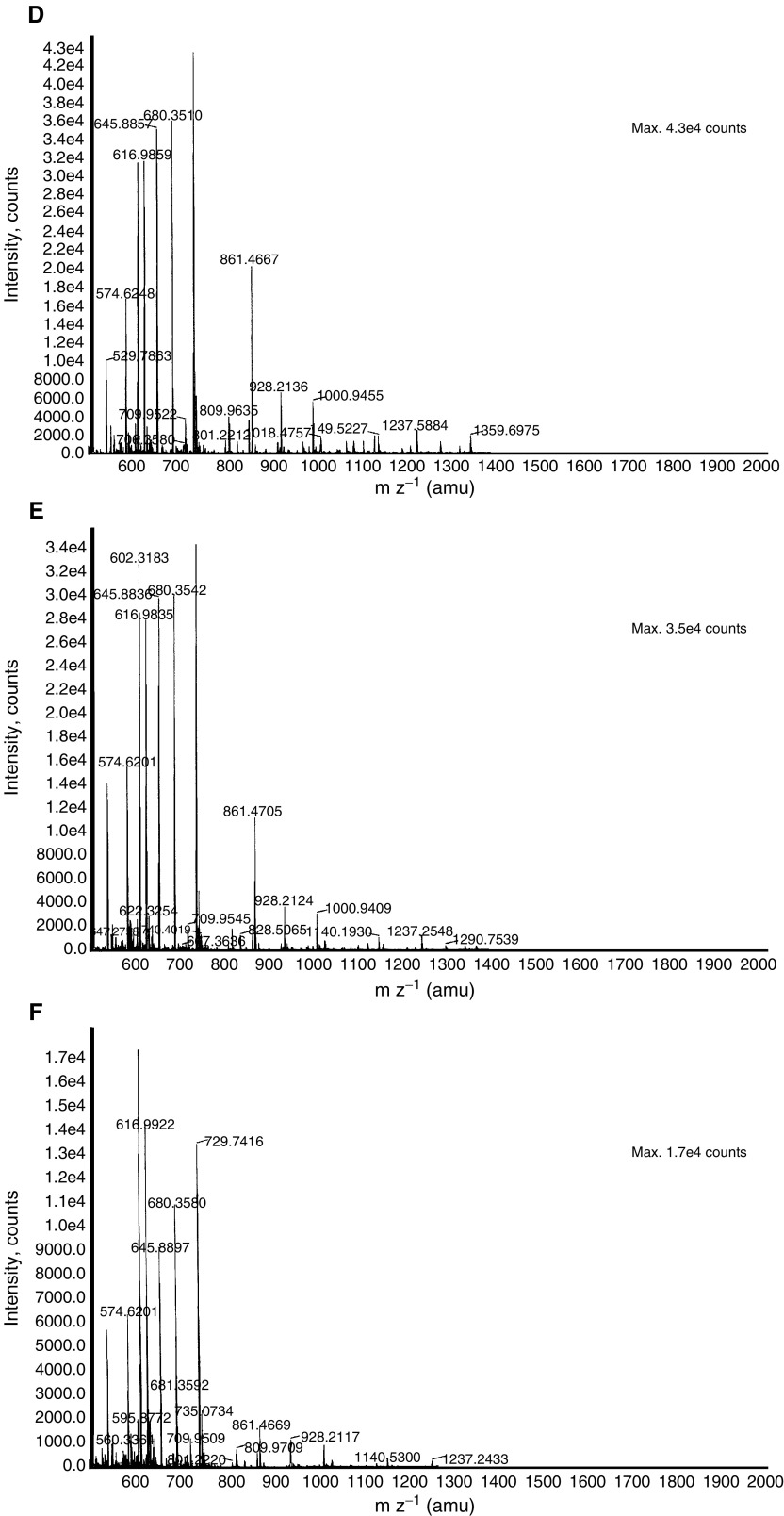
TOFMS spectra obtained for the six HAP1 protein spots. After trypsin digestion, peptide fragments were analysed by n-ESIQ(q)TOFMS in TOFMS and tandem MS mode. (**A**–**F**) Demonstrates the TOFMS spectra of protein spots 1–6. Database searching using the Pep Sea software allowed the identification of the six spots as isoforms of HAP1 (Swissprot accession number P00737). Sequence data are presented in Table 2.

Six protein spots with approximate molecular weights of 40 kDa and pI's 5.9–6.6, all significantly overexpressed in the serum of grade 1, 2 and 3 ovarian cancer patients, were selected for identification and further analysis (Figures 2 and 3

). The protein spots (1–6) could be visualised on the SYPRO-Ruby and Comassie-stained gels. The selected proteins were excised from the gel, subjected to in-gel digestion and analysed by MALDI-TOFMS and n-ESIQ(q)TOFMS. The resultant mass fingerprinting spectra of tryptic digests from the six spots showed identical fragmentation pattern of the peptides, indicating these as series of same protein separating at different pI and/or molecular mass (Figure 4

). Corresponding spectra of the protein was used for protein search in the NCBI and Swissprot database, using the PepSea Server search program or was subjected to MS/MS analysis and then searched through the MASCOT search engine. The six protein isoforms were identified as haptoglobin-1 precursor (HAP1) (NCBI accession number P00737), a protein with a molecular mass of 38.42 kDa and pI of 6.1–6.6. Haptoglobin-1 precursor is more than 90% homologous to circulating haptoglobin, a liver glycoprotein found in normal serum (Beutler *et al*, 2002). Peptide sequence obtained encompassed amino-acid sequence corresponding to different region of HAP1 and is shown in Table 2

**Table 2:** Peptides (underlined) from spots 1–6 corresponding to haptoglobin-1 precursor molecule

MSALGAVIALLLWGQLFAVDSGNDVTDIADDGCPKPPEIA	40
HGYVEHSVRYQCKNYYKLRTEGDGVYTLNNEKQWINKAVG	80
DKLPECEAVCGKPNPANPVQR^103^ILGGHLDAK^111^GSFPWQAKM	120
VSMMNLTTGATLINEQWLLTTAKNLFLNHSENATAK^157^DIAP	160
TLTLYVGK^168^KQ LVEIEKVVLHPNYSQVDIGLIKLNQKVSVN	200
E^202^RVMPICLPSKDYAEVGRV^219^GYVSGWGRNANFKFTDHLK^239^YV	240
MLPVADQDQCIRH^253^YEGSTVPEKKTPK^266^SPVGVQPILNEHTF	280
CRGMSK^286 287^YQEDTCYGDAAGSAFAVHDLEEDTWYATGILSFDK^320^	320
SCAVAEYGVYVKVTSIQDWVQKTIAEN	

**Figure 5:**
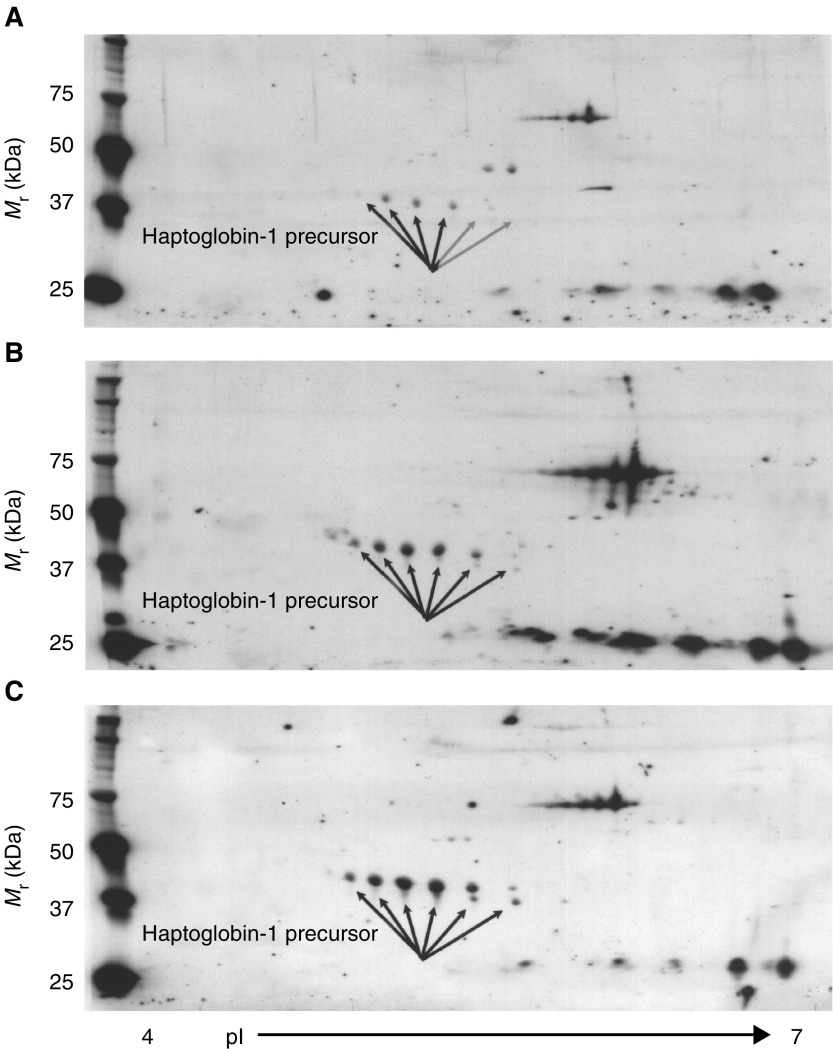
2-DE Western profile of protein spots 1–6 in (**A**) normal, (**B**) grade 1 and (**C**) grade 3 ovarian cancer patients by using monoclonal-anti haptoglobin. Serum samples were resolved for first and second dimensions as described in the Materials and Methods section.

. The identity of HAP1 was further confirmed by 2-DE Western blotting on the serum from healthy volunteers and grade 1 and 3 ovarian cancer patients using monoclonal anti-human haptoglobin antibody that binds to an epitope within HAP1. The antibody recognises both native and denatured human haptoglobin in biological fluids. High immunoreactivity was observed with the set of six proteins at 40 kDa molecular weight by 2-DE Western blot (Figures 5A–C

). The six isoforms of HAP1, exhibited as a chain of protein spots with slightly different molecular mass and different pI's, suggest post-translational modifications.

### Immunohistochemistry analysis of HAP1-like acitivity

Overexpression of proteins on cancer cells can result in their shedding in the peritoneum and hence absorption in the circulation (Sedlaczek *et al*, 2002). We evaluated the expression of HAP1-like immunoreactivity in normal and ovarian cancer tissues. No expression of HAP1-like immunoreactivity was evident in normal ovarian surface epithelium or stroma but some staining was observed in the blood vessels of two cases (*n*=6) (Table 3

**Table 3:** Extent of haptoglobin staining in normal ovarian and tumour tissues

**Grade**	**Case no**	**Clinical stage**	**Haptoglobin staining**
Normal	11		0
	17		0
	19		0
	28		0
	31		0
	37		0
Grade 1	6	1A	3
	51	1B	4
	62	1B	2
	83	1C	2
	106	3C	1
	164	1A	3
	190	1A	2
Grade 2	32	1A	2
	35	3C	2
	46	4	2
	49	NA	3
	110	3C	2
	121	3C	2
	129	3C	4
	161	1C	1
	188	1A	3
Grade 3	2	3C	2
	3	3C	2
	44	2B	2
	90	3C	2
	96	3C	3
	143	4	2
	146	4	3
	153	4	2

The extent of haptaglobin staining in normal ovaries and in tumours was assessed in epithelium, stroma and ovarian cells and scored as 0 (<10%), 1 (11–25%), 2 (26–50%), 3 (51–75%), 4 (76–90%) and 5 (>90%).

NA=not available.

, Figure 6A

**Figure 6:**
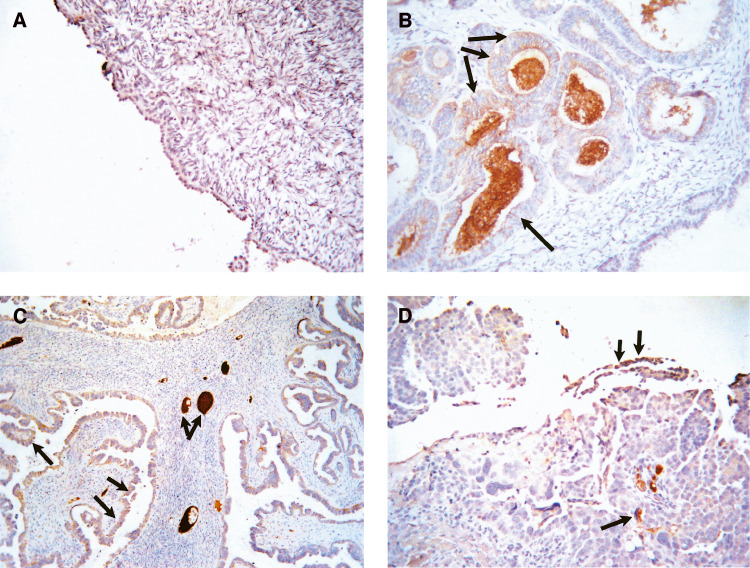
Expression of HAP1-like immunoreactivity in ovarian tissues. Sections of the normal and malignant ovarian tissues were stained by the immunoperoxidase method for the expression of HAP1-like activity as described in the Materials and Methods. (**A**) HAP1-like activity in normal ovary; (**B**) in endometriod grade 1 ovarian tumour, (**C**) serous grade 2 ovarian tumour and (**D**) serous grade 3 ovarian tumour. Arrows indicate the expression of HAP1-like activity in epithelial cells, myxomatous stroma and ovarian vessels.

). However, moderate to strong epithelial staining of HAP1-like reactivity was observed in ovarian tumours (*n*=24, seven grade1, nine grade 2 and eight grade 3) (Table 3, Figure 6B–D). Staining was confined to epithelium, blood vessels and stroma. Cellular staining was mostly cytoplasmic with the majority of the staining being observed in scattered cell groups. Tumours with a glandular pattern tended to have more staining. Strong staining was evident in areas with myxomatous stroma, vascular spaces as well as ovarian vessels. An isotype antibody used as a negative control showed no HAP 1-like staining in normal or tumour tissues.

## DISCUSSION

Better prognosis of ovarian cancer relies on its early detection. Biomarkers are hallmark for cancer detection and the serum proteome of the cancer patient is a rich source of biomarkers due to its modification with disease progression. Within the cancer, cell proteome proteins that are overexpressed by the cells and then released into the bloodstream are ideal markers for early detection and can be used alone or in combination with other tumour markers for screening approaches. Recently, transcriptional profiling or the related serial analysis of gene expression, subtractive hybridisation and differential display technologies have identified 14 candidate ovarian tumour markers (Schummer *et al*, 1999; Mok *et al*, 2001). Among those proteins, mesothelin and kallikrein 10 were identified by monoclonal antibody and candidate gene approaches, respectively (Scholler *et al*, 1999; Luo *et al*, 2001). Mesothelin is elevated in the serum of 76% ovarian cancer patients (Diamandis *et al*, 2000) and kallikrein 10 is elevated in 56% of ovarian cancer patients (Luo *et al*, 2001). Both mesothelin and kallikrein 10 may complement CA 125, increasing the prospect of detecting ovarian cancer at an early, curable stage. Gene array technology has recently identified prostasin (a serine protease, previously identified in prostatic secretions), osteopontin (a secreted bone morphogen) and creatine kinase B (a marker for renal and lung cancers) to be elevated in serum from patients with ovarian cancer (Mok *et al*, 2001; Kim *et al*, 2002). These data indicate that screening approaches at the DNA, RNA or protein level may identify a series of markers that have the potential to complement the currently used marker for more specificity and sensitivity.

Recently, Petricoin *et al* (2002) reported the application of serum proteomic pattern profiling as a diagnostic tool for ovarian cancer. Even though the approach has gained much attention, it is still under evaluation for application to early-stage detection because of its low specificity (∼94%) (Petricoin *et al*, 2002). The approach by Petricon *et al* does not require the identity of individual components of the serum protein for use as a potential diagnostic marker, but focuses mainly on the relative differences of the protein profile of cancer patients compared to healthy volunteers. The present study, however, focuses not only on identifying a proteomic profile of moderate abundance proteins representative of ovarian cancer patients but also attempts to identify ovarian cancer-specific proteins that can be used as biomarkers for early-stage screening.

Even though proteomic approaches have been utilised to discover and identify novel proteins as potential diagnostic/prognostic biomarkers, the technology has been limited by the presence of high abundance proteins such as albumin and immunoglobulin, which can mask or considerably decrease the sensitivity of detection of several low abundant potential biomarkers. Recently, we have demonstrated that the use of Affi-Gel Blue and Aurum kit (Bio Rad Laboratories, USA) results in the removal of highly abundance albumin and simultaneously enhances the detection of several low abundance proteins (Ahmed *et al*, 2003). In this study, we used columns containing a mixture of Affi-Gel Blue and protein A (5 : 1) that is equally effective in removing high abundance albumin. Removal of albumin is achieved with minimal nonspecific protein removal and results in the generation of a protein profile representative of low abundance protein that can be used as a discriminator of a particular disease state. Hence, by using such techniques for sample processing before 2-DE analysis, one can increase the likelihood of discovery of novel biomarkers of high sensitivity and specificity.

Significant differences in the serum protein profiles of grade 1, 2 and 3 ovarian cancer patients (*n*=30) was obtained compared to that of healthy volunteers (*n*=8). Several proteins were found to be differentially or uniquely expressed in the serum of a specific pathological grade of cancer patients and were not consistently expressed among all the histological grades of cancer patients. Compared to healthy volunteers, 24 proteins were differentially expressed in the serum of grade 1 ovarian cancer patients. Grade 2 cancer patients showed differential expression of 31 proteins while that in grade 3 cancer patients 25 proteins were significantly different. Among the differentially expressed serum proteins, 10 common proteins were consistently expressed in grade 1, 2 and 3 cancer patients (*P*<0.05). Six of the common proteins with approximate molecular weights of 40 kDa and pI's 5.9–6.6 were significantly overexpressed in the serum of grade 1, 2 and 3 ovarian cancer patients. These proteins were selected for identification by n-ESIQ(q)TOFMS and MALDI-TOFMS analysis. Mass fingerprinting spectra from the six proteins showed identical fragmentation patterns of the peptides, suggesting the possibility of post-translational modification of a single protein separating at different pI and/or molecular mass. MS/MS analysis of the six proteins confirmed their identity as isoforms of HAP1 (Swissprot accession number P00737), a protein with a molecular mass of 38.42 kDa and pI of 6.1–6.6 that shares 90% homology to a liver glycoprotein haptoglobin, present in the normal serum. Further confirmation of HAP1 was obtained by Western blotting using monoclonal anti-human haptoglobin antibody. The six isoforms of haptoglobin-1 precursor exhibited on 2-DE Western blot as a chain of protein spots with slightly different molecular mass and different pI's.

Haptoglobin is an acute phase protein that binds haemoglobin, thus preventing iron loss and renal damage (Wassell, 2000). The native form of haptoglobin is a ∼90 000 kDa tetramer composed of two nonidentical *α-* and *β*-subunits linked by intermolecular disulphide bonds (Hanley and Heath, 1985). *In vivo* haptoglobin is synthesised as a single polypeptide exhibiting a molecular weight of 38 000 kDa that is proteolytically processed post-translationaly to form the *α-* and *β*-subunits of the native protein (Haugen *et al*, 1981). The precursor protein contains NH_2_-terminal 18 residue signal sequence before the *α* chain and/or intervening polypeptide between the *α-* and *β*-regions (Misumi *et al*, 1983). *In vivo* post-translational event results in the proteolytic removal of the signal sequence and the incorporation of the core oligosaccharide side chains into the *β*-region by membrane-associated enzyme systems (Haugen *et al*, 1981). There is a possibility that post-translation modification also results in the cleavage of both *α-* and *β-*regions of the precursor polypeptide to form the native protein (Haugen *et al*, 1981). The biological implications of the unique mode of biosynthesis and processing of haptoglobin is still not clear but pulse chase experiments have shown that a substantial proportion of the newly synthesised haptoglobin is secreted as a single-polypeptide precursor (Misumi *et al*, 1983). As haptoglobin is among the most abundant glycoproteins secreted by the liver, it is reasonable to hypothesise that enhanced hepatic synthesis of the protein will occur due to an acute phase response in ovarian cancer patients resulting in elevated serum haptoglobin precursor concentration. As most secreted proteins are initially synthesised as larger precursor with an extended NH_2_-terminal sequence that is cleaved at the late-stage secretory process either within the Golgi complex or related vesicles (Schreiber and Urban, 1978), one can assume that the elevation of the HAP1 in cancer patients serum may result due to a disease-specific defective intracellular processing.

Differences in the glycosylation pattern of the protein have the potential to change both the pI and molecular mass of the protein (Wilson *et al*, 2002). Recently, different sialylated forms of haptoglobin have been demonstrated in the normal serum using 2-DE approach (Wilson *et al*, 2002). These observations are consistent with our results demonstrating six different isoforms of HAP1 in the serum of ovarian cancer patients. As the native protein has 90% homology to the precursor, the visualisation of HAP1 on 2-DE Western blot by monoclonal-anti-haptoglobin is not unexpected.

Elevated concentrations of serum haptoglobin were reported in ovarian cancer patients in the early seventies (Mueller *et al*, 1971). Haptoglobin level was shown to be affected by the amount of tumour burden and was not dependent on the histologic type or grade of ovarian malignancy (Mueller *et al*, 1971). Some studies have shown increased fucosylation and other carbohydrate changes in serum haptoglobin of ovarian cancer patients (Thompson *et al*, 1992b). Strong association between abnormally fucosylated haptoglobin and *α* 1,3 fucosyltransferase activity has been demonstrated in the blood specimens of ovarian cancer patients (Thompson *et al*, 1992a). Recently, correlation between haptoglobin, CA 125 and interleukin-6 have been shown in ovarian cancer (Dobryszycka *et al*, 1999). As these studies relied on spectrophotometric (Mueller *et al*, 1971), immunodiffusion (Thompson *et al*, 1992b) and electrophoretic methods (Thompson *et al*, 1992a) for the detection of haptoglobin, it is not surprising that homologous native protein was detected rather than the precursor. Another recent study has shown haptoglobin *α*-subunit as potential serum biomarker in ovarian cancer (Ye *et al*, 2003).

In this study, we also report enhanced expression of HAP1-like activity in ovarian tumours. No immunohistochemical expression of HAP1 was evident in normal ovarian surface epithelium or ovarian stroma. On the other hand, moderate to strong epithelial and stromal staining of HAP1-like activity was observed in ovarian tumours (grades 2 and 3). Epithelial staining was mostly cytoplasmic with majority of the staining being observed in scattered cell groups. Interestingly, strong staining was evident in the myxomatous stroma of malignant ovaries as well as ovarian vessels. These results suggest that HAP1-like expression is present in ovarian tumours but it is the predominant circulating hepatic haptoglobin that is significantly elevated in the serum of ovarian cancer patients.

In conclusion, we present the first evidence that serum HAP1 is significantly increased in early-stage ovarian cancer patients. We suggest that HAP1 may constitute a new and useful biomarker for ovarian cancer diagnosis. These data however need confirmation with further prospective clinical studies.
